# Transplant-free survival in acute liver failure patients receiving MARS®, plasma exchange or no liver support. A real-life 21-year retrospective cohort study in a referral center

**DOI:** 10.1186/s13613-025-01506-3

**Published:** 2025-08-26

**Authors:** Kieran Pinceaux, Félicie Bélicard, Valentin Coirier, Estelle Le Pabic, Pauline Guillot, Flora Delamaire, Benoît Painvin, Quentin Quelven, Mathieu Lesouhaitier, Adel Maamar, Arnaud Gacouin, Pauline Houssel-Debry, Karim Boudjema, Edouard Bardou-Jacquet, Jean-Marc Tadié, Florian Reizine, Christophe Camus

**Affiliations:** 1 Service de Médecine Intensive - Réanimation, CHU de Rennes, 35000 Rennes, France; 2 Centre d’Investigation Clinique, CHU de Rennes, 35000 Rennes, France; 3 Service des Maladies du Foie, CHU de Rennes, 35000 Rennes, France; 4 Service de Chirurgie Hépatobiliaire et Digestive, CHU de Rennes, 35000 Rennes, France

**Keywords:** Acute liver failure, Extracorporeal liver support, Molecular adsorbent recirculating system, Plasma exchange, Liver transplantation

## Abstract

**Background:**

Whether Molecular Adsorbent Recirculating System (MARS) dialysis and high-volume plasma exchange (HVPE) may improve survival in acute liver failure (ALF) remains unclear. A referral center retrospective cohort study was conducted on patients admitted to ICU with ALF and who fulfilled liver transplantation (LTx) criteria from 2000 to 2021.

**Methods:**

The whole study period was divided into three 7-year consecutive periods (A, B, C) depending on the patients’ date of admission. MARS was optionally performed only in periods A and B and HVPE was only performed in period C. Day-21 transplant-free survival (TFS) and day-28 overall survival (OS) were endpoints. The effect of MARS was assessed in periods A and B by comparing the patients treated with MARS with those not treated. Three treatment groups consisting of two different durations of total MARS therapy or no MARS were also compared. HVPE-treated patients (period C, n = 45) were compared to a control group of patients receiving no liver support or a short duration of MARS therapy that was not considered to be effective (over periods A, B, C, n = 126). Survival curves were compared by the Gehan-Breslow-Wilcoxon test and the logrank test.

**Results:**

199 patients were enrolled and distributed as follows: A, n = 68; B, n = 70; C, n = 61. TFS did not differ with and without MARS (p = 0.19). Although MARS duration therapy could not be predicted at the time of MARS initiation, the patients treated ≥ 17 h (≥ 3 sessions) had better survival compared to treatment < 17 h or no MARS (78.6%, 30.4%, 43.8%; p = 0.0002). TFS was 55.6% versus 38.1% in the HVPE- and control groups (p = 0.003; adjusted HR 0.54 [0.32−0.93], p = 0.0257) and OS was 75.9% and 52.9%, respectively (p = 0.03).

**Conclusions:**

MARS therapy improved TFS only in patients who received ≥ 3 sessions. Compared with controls, HVPE-treated patients experienced improved transplant-free and overall survival.

**Supplementary Information:**

The online version contains supplementary material available at 10.1186/s13613-025-01506-3.

## Background

Acute liver failure (ALF) is a rare and life-threatening condition characterized by a sudden decline in hepatic functions. It is defined by elevated transaminases, coagulopathy with prothrombin index (PI) and/or factor V < 50% of the normal ratio and hepatic encephalopathy (HE) evolving over less than 26 weeks, in the absence of pre-existing liver disease [1]. Paracetamol toxicity, ischaemia, drug-induced liver injury, hepatitis B virus, and autoimmunity account for 80% of etiologies in developed countries [1–4]. Because sudden hepatic injury may rapidly evolve into a critically ill state with multiorgan failure, brain edema and ultimately death [4–6], ALF requires prompt admission to the intensive care unit (ICU) [1, 3, 6]. To date, liver transplantation (LTx) remains the gold standard curative treatment for patients with terminal ALF, with a five-year survival rate of up to 70% in Europe [7, 8]. Liver support therapies have been used as a bridge to LTx, or as a bridge to recovery in order to avoid LTx. The Molecular Adsorbent Recirculating System (MARS) is the most widely used device worldwide. Even though prolonged MARS therapy, typically involving ≥ 3 sessions, was associated with better transplant-free survival (TFS) [9, 11, 14], conclusive data from clinical trials are lacking [9–12]. High-volume plasma exchange (HVPE) [15] and standard-volume PE [17] were also reported to improve TFS in ALF. Despite occasional inclusion in international guidelines [18, 19], the benefit of such techniques is not clear in the real-life. The aim of this study was to evaluate the impact of MARS and HVPE, each used for different periods, on the survival of patients with ALF admitted to ICU.

## Materials and methods

### Study design

We conducted a single-center, retrospective, study in the Rennes University Hospital. This hospital is a large volume LTx center and our unit is a referral ICU for the management of ALF patients from a large western area in France (Normandy, Brittany and Pays de la Loire, population of 8 millions inhabitants). From January 1st, 2000 to June 1st, 2021, all patients admitted to the ICU with ALF and who met the usual LTx criteria [20–22] or were listed for LTx were eligible. ALF was defined by the coexistence of elevated transaminases, coagulopathy (PI/factor V < 50%) and HE in patients without preexisting liver disease [1]. Exclusion criteria were: 1) previously known chronic liver disease; 2) admission after LTx for ALF; or 3) refusal to participate. The main endpoint was day-21 TFS defined as the probability of remaining alive and free from LTx. The secondary endpoint was day-28 overall survival (OS). Our study received approval by the Ethics Committee of Rennes University Hospital (n°21.83) and every survivor of the study received written information. 

### General management of ALF in our ICU

All patients had etiological assessment including serum acetaminophen levels, diagnosis for hepatitis viruses, urinary toxicologic screening, auto-immunity, copper dosage, echocardiography and liver imaging [1, 6]. Patients received specific organ support as needed, N-acetyl-cysteine infusion and discontinuation of hepatotoxic drugs. Depending on etiology, specific treatments, such as antivirals, steroids or anticoagulants, were given. Indications for LTx and decision for listing were discussed on a case-by-case basis using usual LTx criteria depending on the ALF etiology (Supplementary Table 1).

### Extracorporeal liver supports

From January 2000 to June 2016, patients could receive MARS dialysis as an adjunct to standard medical therapy (SMT) after individual assessment by clinicians on a case-by-case basis. Because there was no guidelines on the use of MARS in ALF, the decision to initiate MARS therapy in emergency was left to the discretion of the ICU physician in charge of the patient. Since July 1st, 2016, following the results of Larsen’s trial [15], we implemented a new bundle that strongly encouraged the use of HVPE in ALF and MARS dialysis was abandoned [16]. Because this was a new complex protocol, adherence was poor during the first two years. After training sessions with the medical and nursing team, compliance with the protocol became excellent. Finally, all ALF patients received HVPE except for device breakdown, equipment supply problems, unavailability of dedicated staff or moribund patient requiring limited care. The whole study period was divided into 3 chronological periods depending on admission dates: periods A (2000–2007), B (2008–July 2016) and C (August 2016–June 2021). MARS was used only during periods A and B and HVPE was performed in period C. Throughout the study period, MARS and HVPE were realized by a dedicated team specifically trained in extracorporeal therapies (renal replacement therapies [RRT], PE and MARS).

### Data collection

A standardized case report form was used to collect patients’ data including demographic characteristics, and clinical and laboratory parameters at ICU admission and during hospitalization. The severity scores were Glasgow Coma Scale (GCS) score, Simplified Acute Physiology Score II (SAPS II), Sequential Organ Failure Assessment (SOFA) score and Model for End stage Liver Disease (MELD) score. HE severity was graded according to the West Haven criteria [23]. Biological tests included PI, International Normalized Ratio (INR), factor V, fibrinogen, phosphorus, ammonia, creatinine, lactate, pH, bicarbonate, aspartate aminotransferase, alanine aminotransferase, bilirubin, lactate dehydrogenase (LDH). Treatments, organ supports (mechanical ventilation, vasopressors, RRT use, MARS or HVPE) and outcomes (ICU and hospital length of stay, LTx, TFS and OS) were recorded. According to the interval between jaundice and the onset of hepatic encephalopathy, ALF was classified into hyperacute, acute and subacute [2]. ALF etiology was classified into five categories (acetaminophen-related, non-acetaminophen drug-induced or toxic-related, viral, other, indeterminate) and reclassified in two main etiologies: acetaminophen/drug/toxic and non-acetaminophen/drug/toxic.

### Statistical analysis

The statistical analysis was made using Statview® (version 5.0) and SAS® (version 9.4) softwares. Continuous variables were expressed as median (interquartile range [IQR]: 25th percentile-75th percentile) and compared using the Kruskal–Wallis or Mann–Whitney U tests. Categorical variables were reported as numbers and percentages and compared using the Chi-square test or the Fisher’s exact test when appropriate. All tests were two-sided and the statistical significance level was a p-value < 0.05.

Variables associated with day-21 TFS were identified by univariate and multivariate analysis using a Cox model with a stepwise backward selection. The results were reported with Hazard Ratio (HR) of events and 95 percent confidence intervals (95% CI), a HR value < 1 indicating a lower risk of event (improved survival). Survival rates were estimated using the Kaplan–Meier curves and compared by the Gehan-Breslow-Wilcoxon test (day-21 TFS) or the logrank test (day-28 OS). To assess the effect of HVPE, the treated group was compared to a control group comprising all the patients who received either no liver support or an insufficient duration of MARS, considered ineffective [13, 14]. Survival analysis was adjusted for prognostic variables, including ALF etiology, N-acetyl-cysteine treatment, HE severity, INR, lactate levels, SOFA score and organ failure support.

## Results

### Patient characteristics at baseline and clinical course in ICU

**Fig. 1:**
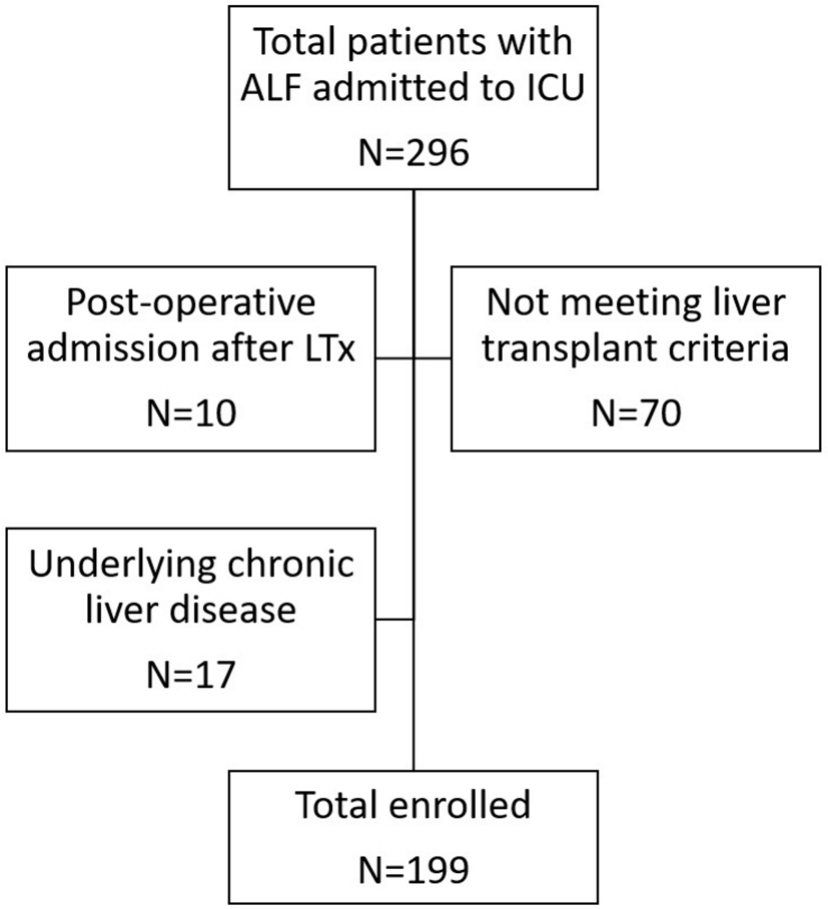
Patient selection flowchart. *ALF* Acute Liver Failure, *ICU* Intensive Care Unit, *LTx* Liver transplantation

Among the 296 patients admitted to ICU with ALF, 70 did not meet transplant criteria and 27 had exclusion criteria. Finally, 199 patients were included in the study (flowchart, Fig. 1). They were distributed in 68, 70 and 61 patients for periods A, B and C respectively. Baseline characteristics at admission to the ICU are shown in Supplementary Table 2. Patients were predominantly female (52.3%) with a median age of 47 years (35–57). Clinical characteristics were mostly similar between periods, except for neurological history, prior immunosuppressive therapy and use of RRT during the first 24 h in ICU. Biological features at admission were mostly identical, except for INR, creatinine, LDH and MELD score. All five etiologies were equally distributed during the periods although acetaminophen-related ALF tended to increase over time (χ^2^-test for trend, p = 0.0129). Patients course in ICU and hospital stay is described in Supplementary Table 3. ALF was classified into hyperacute (n = 167, [83.9%]), acute (n = 20, [10.1%]) and subacute (n = 12, [6.0%]), without difference between periods. One hundred percent of paracetamol-related ALF, 93% of toxic-non-paracetamol ALF (41/44) and 78% of viral ALF (14/18) were hyperacute. There were some differences regarding the worst values of INR, phosphorus, pH, LDH and the highest MELD score. Treatment and outcomes did not differ over time except for the use of N-acetyl-cysteine and specific etiological treatment and the registration on the transplant list.

### Effect of MARS

**Fig. 2:**
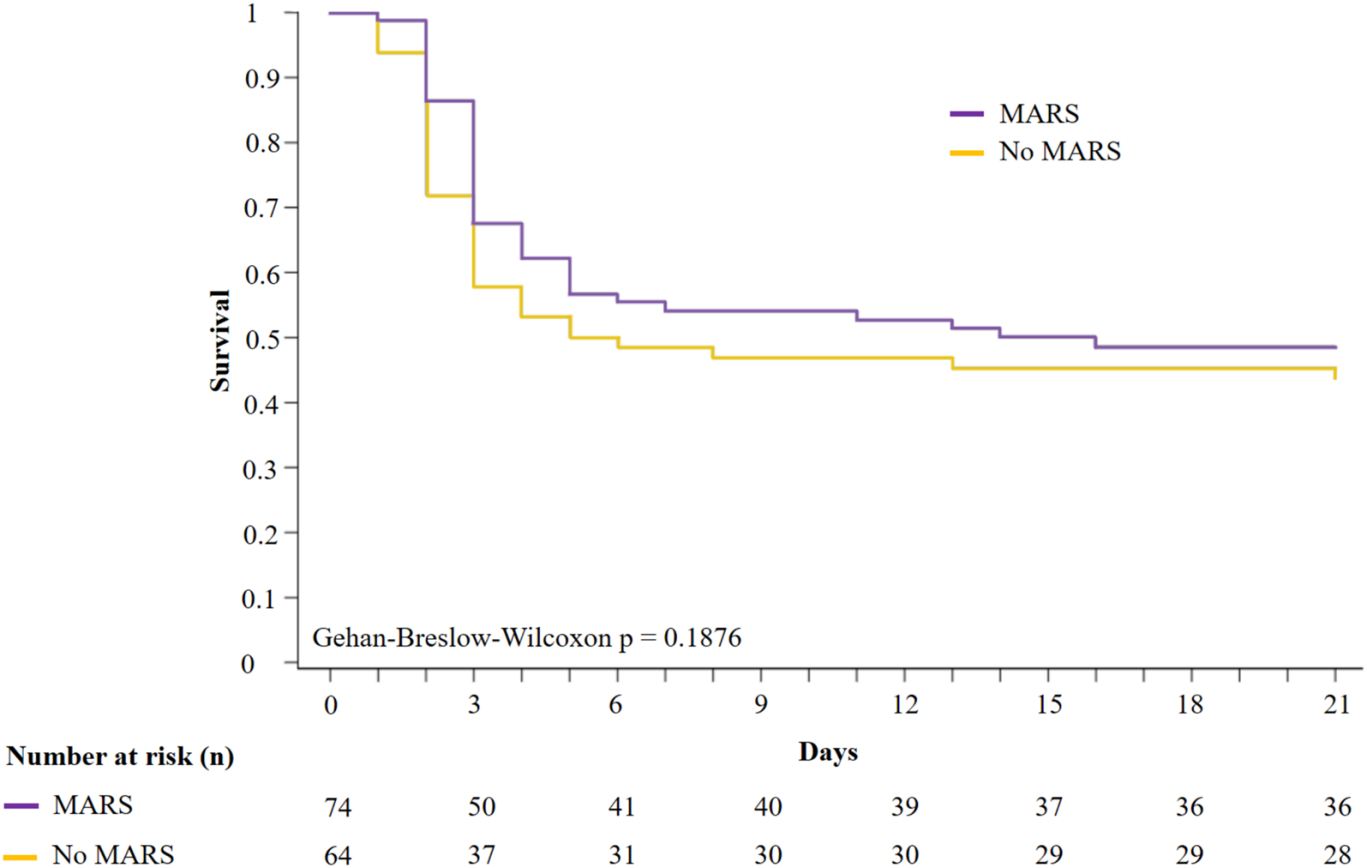
Day-21 transplant-free survival among patients in periods A and B depending on MARS therapy. *MARS* Molecular Adsorbent Recirculating System

Among the 138 patients in periods A and B, 74 (53.6%) received at least one session of MARS dialysis. The elapsed time between ICU admission and the first session was 12 h [7–19], with a median of 2 sessions per patient [1–3]. The session duration was 8 h [6.8–8] and the total duration of MARS therapy was 15.5 h [8–24]. Baseline characteristics and ICU course are detailed in Supplementary Table 4 and were compared depending on MARS therapy. There was no significant difference between groups except for the SOFA score at admission (p = 0.0286), as well as the respiration and coagulation SOFA subscores (p = 0.0239 and p = 0.0159 respectively). Day-21 TFS did not differ between groups (48.6% in the MARS group and 43.8% in the no-MARS group, p = 0.1876) (Fig. 2). Analysis of day-21 TFS is shown in Table 1. By univariate analysis, MARS was not associated with improved TFS (p = 0.37) or OS (p = 0.32). In multivariate analysis, acetaminophen or toxic etiology, improvement of HE within the first 24 h in ICU and therapy by N-acetyl-cysteine were associated with a higher day-21 TFS, and HE of grade 3–4 (or intubation), INR > 6.5 and lactate > 5 mmol/L at admission with a lower day-21 TFS.

**Table 1:** Univariate and multivariate analysis of Day-21 transplant-free survival among the 138 patients in periods A and B

	Univariate analysis		Multivariate analysis	
Variables	HR [CI 95%]	p-value	HR [CI 95%]	p-value
Obesity	3.28 [1.70–6.32]	0.0004		
Acetaminophen or toxic etiology	0.38 [0.24–0.61]	< 0.0001	0.54 [0.30–0.97]	0.0397
SOFA score > 8 at admission	1.70 [1.07–2.70]	0.0247		
SAPS II > 50 at admission	2.53 [1.56–4.12]	0.0002		
MELD > 38 at admission	2.31 [1.43–3.73]	0.0006		
Grade 3–4 (or intubation) HE at admission	1.85 [0.96–3.59]	0.028	2.14 [1.06–4.34]	0.0274
Need for vasopressors during the first 24 h	2.36 [1.49–3.74]	0.0002		
Need for mechanical ventilation during the first 24 h	2.10 [1.31–3.38]	0.0022		
Need for renal replacement therapy during the first 24 h	1.89 [1.20–2.99]	0.0064		
Improvement of HE within 24 h after admission	0.16 [0.06–0.40]	< 0.0001	0.16 [0.06–0.42]	0.0001
INR > 6,5 at admission	1.90 [1.20–3.01]	0.0059	1.82 [1.10–3.00]	0.0190
Lactate > 5 mmol/L at admission	2.56 [1.58–4.16]	0.0001	1.78 [1.06–3.00]	0.0296
Use of MARS therapy	0.81 [0.51–1.28]	0.3721		
Immunosuppressive therapy during the past year	1.88 [1.03–3.43]	0.0385		
Use of N-acetyl-cysteine	0.37 [0.23–0.60]	< 0.0001	0.51 [0.28–0.92]	0.0247

*SOFA* Sequential Organ Failure Assessment, *SAPS II* Simplified Acute Physiology Score II, *MELD* Model for End stage Liver Disease, *HE* Hepatic Encephalopathy, *INR* International Normalized Ratio, *MARS* Molecular Adsorbent Recirculating System

The total number of MARS sessions and the total duration of MARS were associated with improved day-21 TFS (HR per MARS session 0.58 [0.41–0.81], p = 0.0016; HR per hour of MARS 0.94 [0.90–0.97], p = 0.009). Sensitivity analysis found that the best threshold for predicting day-21 TFS was a total duration of MARS therapy ≥ 17 h. Among the 74 patients treated with MARS, 46 (62.2%) received ≥ 17 h and 28 (37.8%) received < 17 h. Baseline characteristics and outcomes of both subgroups were compared in Supplementary Table 5. There was no significant difference at baseline. The proportion of patients with contraindications to LTx was 19.6% and 46.4% in the groups receiving < 17 h and ≥ 17 h of MARS, respectively (p = 0.0142). The number of sessions per patient and the total duration of MARS therapy was 1 [1, 2], 8 h [7.5–13.5] and 3 [3, 4], 24 h [22.25–32] in the < 17 h and ≥ 17 h of MARS groups, respectively (p < 0.0001). The variables associated with day-21 TFS among the 74 patients treated with MARS are shown in Supplementary Table 6. HE improvement within the first 24 h, N-acetyl-cysteine therapy and receiving ≥ 17 h of MARS were associated with a higher day-21 TFS, and admission MELD score > 38 and vasopressors during the first 24 h with a lower day-21 TFS.

**Fig. 3:**
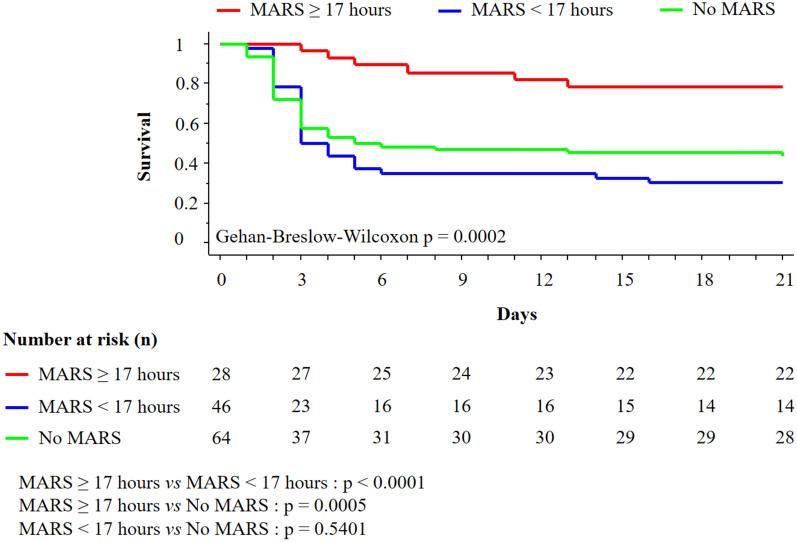
Day-21 transplant-free survival among patients in periods A and B according to MARS therapy duration. *MARS* Molecular Adsorbent Recirculating System

Survival was assessed in three groups of patients in periods A and B. TFS was 78.6% in the group receiving ≥ 17 h of MARS, 30.4% in the group receiving < 17 h of MARS and 43.8% in the group without MARS (p = 0.0002, Fig. 3). There was no significant difference between the group receiving < 17 h of MARS and the group without MARS (p = 0.5401).

**Fig. 4:**
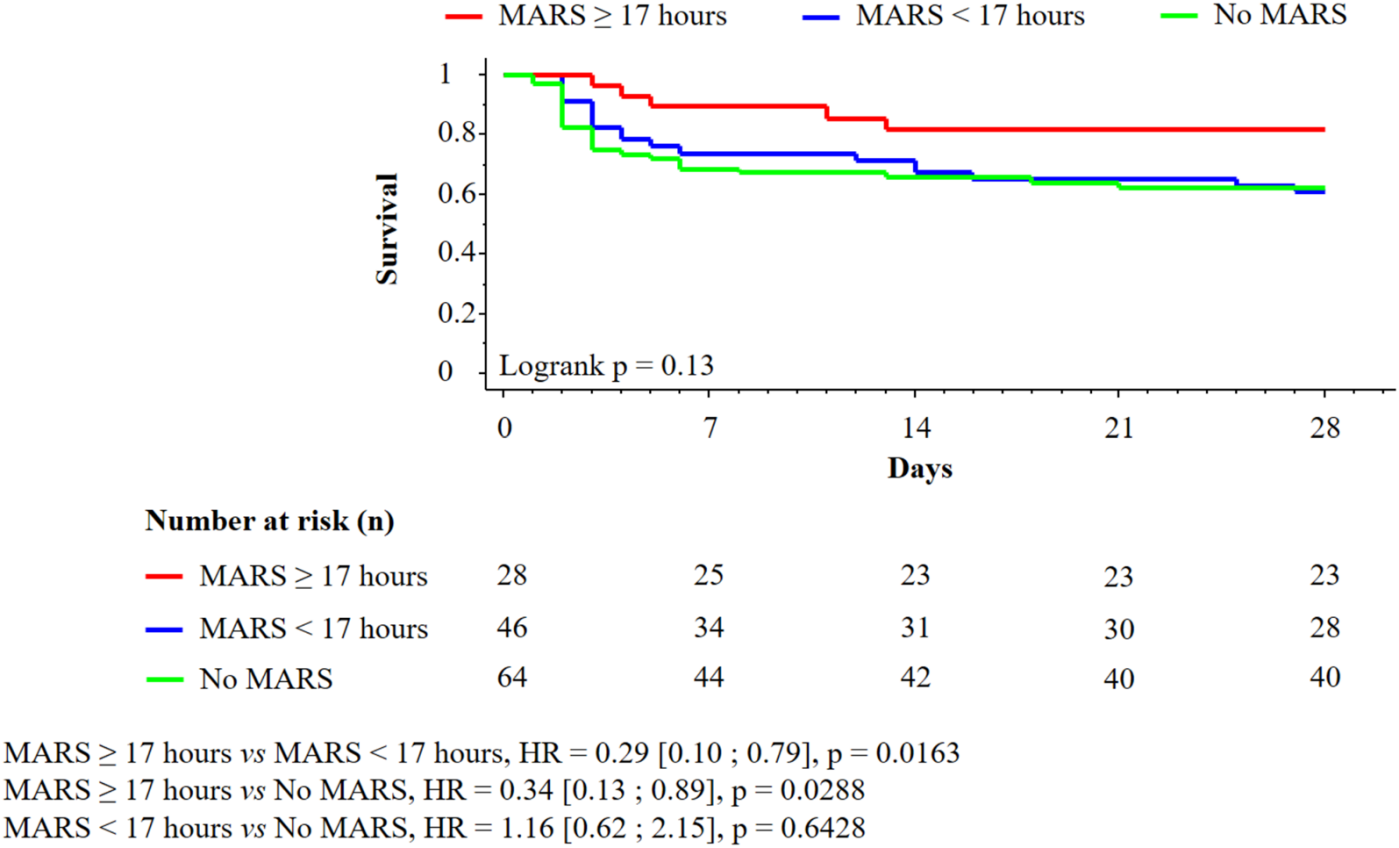
Day-28 overall survival among patients in periods A and B according to MARS therapy duration. *MARS* Molecular Adsorbent Recirculating System

The day-28 OS was 82.1% in the group receiving ≥ 17 h of MARS, 60.9% in the group receiving < 17 h of MARS and 62.5% in the group without MARS (p = 0.13, Fig. 4). The day-28 OS was higher in the group receiving ≥ 17 h of MARS compared to the group receiving < 17 h of MARS (after stratification for LTx, HR 0.29 [0.10–0.79], p = 0.0163).

### Effect of HVPE

**Fig. 5:**
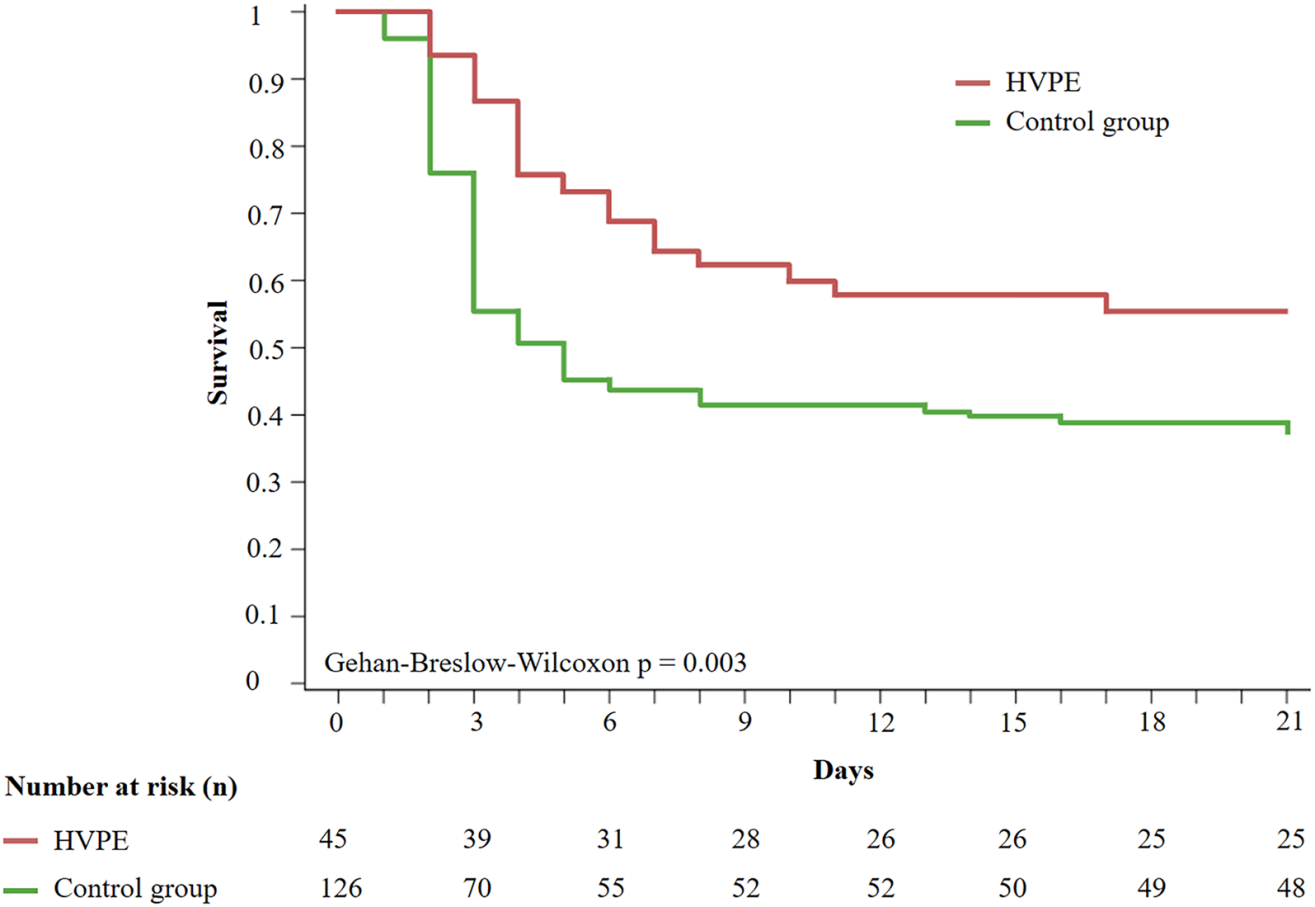
Day-21 transplant-free survival in 45 patients treated with HVPE and in 126 controls. *HVPE* High Volume Plasma Exchange

Forty-five patients received HVPE treatment. They were compared to the 126 patients of the control group, who received either no liver support or MARS therapy < 17 h (2 or 1 session). Those who received ≥ 17 h (≥ 3 sessions) of MARS were excluded from the control group. Among the HVPE group, the time from ICU admission to the first session was 9 h [4.65–21], with a median number of sessions per patient of 3 [2, 3]. Seven (15.6%), 10 (22.2%) and 28 (62.2%) patients received one, two and three sessions, respectively. Baseline characteristics, course and outcomes of the 45 HVPE patients and 126 controls are detailed in Supplementary Table 7. Both groups were comparable, except for more frequent prior immunosuppression, higher baseline SOFA score, more frequent RRT within the first 24 h and less frequent use of N-acetyl-cysteine in the control group. In the crude analysis, the day-21 TFS was 55.6% in the HVPE group and 38.1% in the control group (p = 0.003, Fig. 5). After adjustment for confounding factors (HE grade, SOFA score, need for RRT within the first 24 h, immunosuppressive therapy, N-acetyl-cysteine treatment, ALF etiology, SAPS II, INR, worsening of HE after admission), day-21 TFS remained higher in the HVPE group (HR 0.54 [0.32–0.93], p = 0.0257). The day-28 OS was 75.6% in the HVPE group and 59.5% in the control group (logrank, stratification for LTx, p = 0.03, Supplementary Fig. 1). Among the 121 patients without LTx contraindications (31 in the HVPE group and 90 in the control group), day-21 TFS was higher in the HVPE-treated compared to controls (61.3% *vs* 46.7%, p = 0.0150, Supplementary Fig. 2). Among patients not listed for LTx, the day-28 OS was higher in the HVPE group (75.9% *vs* 52.9%, p = 0.0364, Supplementary Fig. 3). Among those not listed due to contraindications, the day-28 OS was 42.9% in the HVPE group and 16.7% in the control group (p = 0.0583, Supplementary Fig. 4).

## Discussion

Our study aimed to assess the effect of MARS dialysis and HVPE on survival outcomes in patients with severe ALF meeting transplant criteria. The baseline characteristics of our cohort align with existing literature data and the prognosis factors identified by multivariate analysis were consistent with previous studies [2–4, 20].

Our findings indicated that the use of MARS did not improve survival outcomes, in agreement with the results of one randomized clinical trial conducted by Saliba et al*.* (FULMAR study) [9]. Importantly, our study evaluated nearly twice more patients treated with MARS, with a lower proportion of patients undergoing LTx in our study (23.9% *vs* 64.7%). However, we found a relationship between the duration of MARS and TFS. Patients receiving ≥ 17 h of MARS therapy (in practice, 3 sessions or more) had a better day-21 TFS, suggesting that a minimum of 3 sessions are required to achieve survival improvement, in accordance with prior studies [9, 11, 14]. We were not able to predict the duration of MARS therapy at the time of initiation, since we found no difference in baseline characteristics between the patients treated < 17 h and those treated ≥ 17 h (Supplementary Table 5). Due to the retrospective design of our study, it was not possible to fully explain the decision to MARS dialysis. Many factors could have interfered with the initiation and the duration of MARS: logistical problems related to dedicated staff or MARS device, contraindication to MARS (hemodynamic instability, very low fibrinogen or platelet count), premature coagulation of the circuit or filter clogging, intra-dialytic disseminated intravascular coagulation, hypotension and hypothermia [9, 10, 24]. In patients listed for “superurgence” LTx, the time between the initiation of MARS dialysis and the availability of a liver graft was an important competing factor for the total duration of MARS therapy. The time between listing and graft proposal could be very short (< 24 h) in the French allocating system and precipitate the interruption of MARS sessions generally performed on a daily schedule. Because nearly half of the patients receiving MARS for ≥ 17 h had contraindications to LTx, MARS therapy could better target ALF patients who are not suitable for LTx.

Regarding the effect of HVPE, our results showed that day-21 TFS was improved in patients treated with HVPE as compared with controls (HR 0.54 [0.32–0.93], p = 0.0257). This finding was consistent with the results of Larsen et al. (HR = 0.56 [0.36–0.86], p = 0.0083) [15]. In our study, the beneficial effect of HVPE was also observed in day-28 OS (p = 0.03) and in patients without LTx contraindications (p = 0.0150). Additionally, although statistical significance was not reached, our results also showed a benefit of HVPE on survival in patients not listed due to contraindication to LTx (42.9% *vs* 16.7%, p = 0.0583), similar to the results of Larsen et al*.* in the same subgroup of patients (36% *vs* 20%, p = 0.03). Altogether, these data indicate that HVPE could be used not only for patients who are contraindicated to LTx or in settings with limited access to grafts, but also for those on the waiting list. Whether the efficacy of PE is related to the high volume substituted is unclear. A randomized controlled study using standard-volume plasma exchange (SVPE) *vs* SMT in ALF patients with no absolute contraindication to LTx also found a benefit on TFS [17]. More recently, Kulkarni et al. suggested that HVPE and SVPE had similar efficacy [25].

Our study has several limitations. First, due to its single-center and retrospective design, the conclusions are not necessarily generalizable to other centers. Second, the potential benefit of prolonged MARS therapy is limited by the impossibility to predict the total treatment duration at the time of MARS initiation. Third, the extended inclusion period of the control group over 21 years implies potential changes in practices and recruitment in the ICU. This limits the comparability between the HVPE group and the control group, emphasizing the need for cautious interpretation of our findings. However, our study reflected the real-world use of two invasive extracorporeal liver support modalities, administered by a specialized team in a tertiary-referral ICU. Because ALF is a rare disease, the size of our cohort is significant and may provide useful insights into clinical practice. Given the complexity of conducting randomized controlled trials assessing extracorporeal liver support devices in ALF, retrospective series like ours may offer valuable evidence on the effect of MARS therapy and HVPE.

## Conclusions

In ALF patients, MARS dialysis improved TFS only in those who could receive three complete sessions or more. Because LTx could compete with MARS dialysis, this therapy could best target patients with contraindications to LTx. Compared with a historical control group, HVPE-treated patients had both improved TFS and OS. As there is growing evidence for survival benefit with PE to treat ALF, HVPE could be used not only in patients contraindicated for LTx but also in those without contraindications, as a bridge to recovery or second-step LTx. The interest of MARS combined with PE deserves further investigation.

## Supplementary Information

Additional file 1.

## Data Availability

All data generated or analysed during this study are included in this published article and its supplementary information files.

## Abbreviations

MARS: Molecular adsorbent recirculating system
HVPE: High-volume plasma exchange
ALF: Acute liver failure
LTx: Liver transplantation
TFS: Transplant-free survival
OS: Overall survival
PI: Prothrombin index
HE: Hepatic encephalopathy
ICU: Intensive care unit
SMT: Standard medical therapy
RRT: Renal replacement therapies
GCS: Glasgow Coma Scale
SAPS II: Simplified acute physiology score II
SOFA: Sequential organ failure assessment
MELD: Model for end stage liver disease
INR: International normalized ratio
LDH: Lactate dehydrogenase
IQR: Interquartile range
HR: Hazard ratio
SVPE: Standard-volume plasma exchange
