# Correction: Transplant-free survival in acute liver failure patients receiving MARS^®^, plasma exchange or no liver support. A real-life 21-year retrospective cohort study in a referral center

**DOI:** 10.1186/s13613-025-01574-5

**Published:** 2025-10-10

**Authors:** Kieran Pinceaux, Félicie Bélicard, Valentin Coirier, Estelle Le Pabic, Pauline Guillot, Flora Delamaire, Benoît Painvin, Quentin Quelven, Mathieu Lesouhaitier, Adel Maamar, Arnaud Gacouin, Pauline Houssel-Debry, Karim Boudjema, Edouard Bardou-Jacquet, Jean-Marc Tadié, Florian Reizine, Christophe Camus

**Affiliations:** 1 Service de Médecine Intensive - Réanimation, CHU de Rennes, 35000 Rennes, France; 2 Centre d’Investigation Clinique, CHU de Rennes, 35000 Rennes, France; 3 Service des Maladies du Foie, CHU de Rennes, 35000 Rennes, France; 4 Service de Chirurgie Hépatobiliaire et Digestive, CHU de Rennes, 35000 Rennes, France

**Correction: Annals of Intensive Care (2025) 15:124** 10.1186/s13613-025-01506-3

In the sentence beginning in the Background section “Even though prolonged MARS therapy, typically involving ≥ 3 sessions, was associated with better transplant-free survival (TFS) [9, 11, 14], conclusive data from clinical trials are lacking [9−12]”, the symbol between 11 and 14 should have been “−” instead of “,”. The correct sentence should be “Even though prolonged MARS therapy, typically involving ≥ 3 sessions, was associated with better transplant-free survival (TFS) [9, 11−14], conclusive data from clinical trials are lacking [9−12]”.

In the sentence beginning in the Results section “Among the 74 patients treated with MARS, 46 (62.2%) received ≥ 17 h and 28 (37.8%) received < 17 h”, the symbols ≥ and < were interchanged, leading to misinterpretation for the reader. The correct sentence should be “Among the 74 patients treated with MARS, 46 (62.2%) received < 17 h and 28 (37.8%) received ≥ 17 h”.

