# Supplementary material for: Transplant-free survival in acute liver failure patients receiving MARS®, plasma exchange or no liver support. A real-life 21-year retrospective cohort study in a referral center
Source: Ann Intensive Care. 2025 Aug 26;15:124. doi: 10.1186/s13613-025-01506-3 (PMC12381347; doi:10.1186/s13613-025-01506-3)
Supplement: Supplementary file 1 — Additional file 1. [file 13613_2025_1506_MOESM1_ESM.docx]

**Supplementary Table 1. Poor prognosis criteria indicating liver transplantation**

| Clichy criteria (any etiology)^1,2^ | Age less than 30 years  Confusion or coma (grade 3 or grade 4 hepatic  encephalopathy) AND  factor V <20% |
| --- | --- |
|  | Age ≥ 30 years  Confusion or coma (grade 3 or grade 4 hepatic  encephalopathy) AND  factor V < 30% |
| King’s College criteria : paracetamol^3,4^ | 1. Arterial pH is below 7.3 OR 2. Arterial lactate concentration is above 3.0 mmol/L after adequate fluid resuscitation OR |
|  | 1. concurrently, |
|  | - Serum creatinine is above 300 µmol/L (or AKI Kdigo 3) - INR is above 6.5 - Hepatic encephalopathy of grade 3 or greater |
| King’s College criteria : non paracetamol^3^ | 1. INR >6.5, irrespective of grade of encephalopathy OR |
|  | 1. Three or more of the following variables, irrespective of grade of encephalopathy* |
|  | - Age >40 years - “Unfavorable” etiology (drug-induced hepatitis, indeterminate…) - Duration of jaundice before onset of encephalopathy >7 days - INR >3.5 - Serum bilirubin >300 µmol/L |
| Escudie criteria: amatoxin mushroom poisoning^5^ | From day 4 after ingestion,  prothrombin index lower than 10% (INR >6) |

NOTE. 1 Bernuau J, AASLD Abstracts, Hepatology 1991. 2 Bismuth H, Ann Surg 1995. 3 O’Grady JG, Gastroenterology 1989. 4 Bernal W, Lancet 2002. 5 Escudié L, J Hepatol 2007. * Adapted from [3] for our ICU population. Confusion was required for transplantation.

REFERENCES

1 - Bernuau J et al. Criteria for emergency liver transplantation in patients with acute viral hepatitis and factor V (FV) below 50% of normal: a prospective study. He AASLD Abstracts. Hepatology 1991;14(No. 4, Pt 2):49A.

2 - Bismuth H et al. Orthotopic liver transplantation in fulminant and subfulminant hepatitis. The Paul Brousse experience. Ann Surg 1995 Aug;222(2):109-19. doi: 10.1097/00000658-199508000-00002.

3 - O'Grady JG, Alexander GJ, Hayllar KM, Williams R. Early indicators of prognosis in fulminant hepatic failure. Gastroenterology. 1989 Aug;97(2):439-45. doi: 10.1016/0016-5085(89)90081-4.

4 - Bernal W, Donaldson N, Wyncoll D, Wendon J. Blood lactate as an early predictor of outcome in paracetamol-induced acute liver failure: a cohort study. Lancet. 2002 Feb 16;359(9306):558-63. doi: 10.1016/S0140-6736(02)07743-7.

5 - Escudié L, et al. Amanita phalloides poisoning: reassessment of prognostic factors and indications for emergency liver transplantation. J Hepatol. 2007 Mar;46(3):466-73. doi: 10.1016/j.jhep.2006.10.013.

**Supplementary table 2: Baseline characteristics at admission in intensive care unit according to the inclusion period**

|  | | | | **Periods** | | |  |
| --- | --- | --- | --- | --- | --- | --- | --- |
| **Variables** | | | **Total (n = 199)** | **A (n = 68)** | **B (n = 70)** | **C (n = 61)** | **P value** |
| Sex M/F (no. (%)) | | | 95/104 (47.7/52.3) | 36/32 (52.9/47.1) | 31/39 (44.3/55.7) | 28/33 (45.9/54.1) | 0.5614 |
| Age (years) | | | 47 (35-57) | 45 (37-55) | 47 (33-56) | 49 (37-59) | 0.5979 |
| SOFA score | | | 8 (5-12) | 8 (5-12) | 9 (5-14) | 7 (4-11) | 0.1472 |
| SAPS II score | | | 47 (31-65) | 46 (30-65) | 52 (35-70) | 45 (29-58) | 0.1829 |
| Glasgow Coma Scale | | | 14 (8-15) | 14 (10-15) | 14 (8-15) | 14 (4-15) | 0.2463 |
| Prothrombin rate (%) | | | 16 (11-24) | 14 (11-23) | 19 (13-25) | 13 (6-24) | 0.1249 |
| INR* | | | 5.3 (3.5-9.7) | 8.1 (4.5-10.9) | 4.1 (3.1-6.2) | 5.5 (2.9-9) | 0.0001 |
| Factor V (%) | | | 16 (10-24) | 17 (11-24) | 16 (7-23) | 16 (11-27) | 0.4823 |
| Fibrinogen (g/L) | | | 1.6 (1.1-2.2) | 1.5 (1-2) | 1.6 (1.2-2.2) | 1.7 (1.2-2.2) | 0.4923 |
| Phosphorus (mmol/L) | | | 1.01 (0.69-1.59) | 0.9 (0.66-1.37) | 1.06 (0.62-1.69) | 1.08 (0.76-1.6) | 0.4504 |
| Ammonium (µmol/L) | | | 104 (66-144) | 101 (65-141) | 96 (60-148) | 106 (74-146) | 0.6598 |
| Creatinine (µmol/L) | | | 145 (74-261) | 154 (84-303) | 186 (72-295) | 117 (57-173) | 0.0076 |
| Lactate (mmol/L) | | | 5 (2.6-9.5) | 4.6 (2.6-10.8) | 5.2 (2.6-8.8) | 5.1 (2.9-9.2) | 0.8175 |
| pH | | | 7.39 (7.28-7.47) | 7.41 (7.32-7.47) | 7.38 (7.25-7.45) | 7.36 (7.29-7.45) | 0.091 |
| Bicarbonate (mmol/L) | | | 20 (15-25) | 21 (15-25) | 20 (15-27) | 19 (15-23) | 0.6975 |
| ASAT (UI/L) | | | 4331 (1406-10400) | 4016 (1348-9920) | 5847 (1600-11100) | 3771 (1301-9054) | 0.2171 |
| ALAT (UI/L) | | | 3415 (1436-5059) | 2667 (1277-4398) | 3688 (1445-6392) | 3644 (1494-4895) | 0.4004 |
| Total bilirubin (µmol/L) | | | 87 (45-158) | 99 (51-164) | 85 (43-139) | 81 (42-146) | 0.2906 |
| Conjugated bilirubin (µmol/L) | | | 53 (29-104) | 58 (34-128) | 48 (28-86) | 65 (28-106) | 0.4382 |
| LDH (UI/L) | | | 4253 (1204-10046) | 4080 (1204-9703) | 7302 (2094-11042) | 2776 (799-7065) | 0.0083 |
| MELD score* | | | 38 (30-44) | 41 (33-50) | 36 (29-42) | 37 (28-41) | 0.0035 |
| Medical history (no. (%)) | | |  | | | | |
|  | Arterial hypertension | | 11 (16.2) | 11 (15.7) | 6 (9.8) | 28 (14.0) | 0.5193 |
|  | Diabetes mellitus | | 9 (4.5) | 1 (1.5) | 4 (5.7) | 4 (6.6) | 0.3195 |
|  | Obesity | | 14 (7.0) | 8 (11.8) | 3 (4.3) | 3 (4.9) | 0.1692 |
|  | Cardiovascular disease | | 22 (11.0) | 6 (8.8) | 11 (15.7) | 5 (8.2) | 0.3017 |
|  | Neurological disease | | 23 (11.6) | 14 (20.6) | 6 (8.6) | 3 (4.9) | 0.0131 |
|  | Renal disease | | 6 (3.0) | 3 (4.4) | 3 (4.3) | 0 (0.0) | 0.2546 |
|  | Cancer/hemopathy | | 22 (11.0) | 10 (14.7) | 9 (12.9) | 3 (4.9) | 0.1747 |
|  | Immunosuppression | | 31 (15.6) | 16 (23.5) | 11 (15.7) | 4 (6.6) | 0.0295 |
|  |  | HIV | 3 (1.5) | 1 (1.5) | 2 (2.9) | 0 (0.0) | 0.3597 |
|  |  | Immunosuppressive therapy during the past year | 16 (8.0) | 11 (16.2) | 5 (7.1) | 0 (0.0) | 0.0032 |
| Active infection (no. (%)) | | | 80 (40.2) | 33 (48.5) | 25 (35.7) | 22 (36.0) | 0.2251 |
| Mechanical ventilation (no. (%)) | | | 54 (27.1) | 15 (22.1) | 23 (32.9) | 16 (26.2) | 0.2551 |
| Mechanical ventilation during the first 24 hours (no. (%)) | | | 101 (50.8) | 31 (45.6) | 39 (55.7) | 31 (50.8) | 0.4928 |
| Renal replacement therapy (no. (%)) | | | 3 (1.5) | 0 (0.0) | 2 (2.9) | 1 (1.6) | 0.3855 |
| Renal replacement therapy during the first 24 hours (no. (%)) | | | 71 (35.7) | 27 (39.7) | 31 (44.3) | 13 (21.3) | 0.0164 |
| Vasopressors (no. (%)) | | | 37 (18.6) | 8 (11.8) | 15 (21.4) | 14 (23.0) | 0.1987 |
| Vasopressors during the first 24 hours (no. (%)) | | | 74 (37.2) | 22 (32.4) | 29 (41.4) | 23 (37.7) | 0.5416 |
| HE grade (West-Haven criteria) | | |  |  |  |  | 0.1919 |
|  | 0 | | 45 (22.6) | 11 (16.2) | 14 (20.0) | 20 (32.8) |  |
|  | 1 - 2 | | 64 (32.2) | 25 (36.8) | 21 (30.0) | 18 (29.5) |  |
|  | 3 - 4 (or intubation) | | 90 (45.2) | 32 (47.0) | 35 (50.0) | 23 (37.7) |  |
| ALF etiologies | | |  |  |  |  | 0.1387 |
|  | Acetaminophen | | 82 (41.2) | 20 (29.4) | 31 (44.3) | 31 (50.8) |  |
|  | No-acetaminophen toxicity | | 44 (22.1) | 17 (25.0) | 17 (24.3) | 10 (16.4) |  |
|  | Virus | | 18 (9.0) | 5 (7.4) | 6 (8.6) | 7 (11.5) |  |
|  | Others | | 36 (18.1) | 16 (23.5) | 13 (18.6) | 7 (11.5) |  |
|  | Indeterminate | | 19 (9.5) | 10 (14.7) | 3 (4.3) | 6 (9.8) |  |

Data are expressed as median (25^th-^75^th^ percentiles) or numbers (%). Continuous variables are compared by the Mann-Whitney U test or the Kruskal-Wallis test and categorical variables are compared by the χ² test or the Fisher’s exact test. A P value < 0.05 is considered significant. ICU: Intensive Care Unit, SOFA: Sequential Organ Failure Assessment, SAPS II: Simplified Acute Physiology Score II, INR: International Normalized Ratio, ASAT: Aspartate aminotransferase, ALAT: Alanine aminotransferase, LDH: Lactate Dehydrogenase, MELD: Model for End stage Liver Disease, HIV: Human Immunodeficiency Virus, HE: Hepatic Encephalopathy, ALF: Acute Liver Failure. *In periods B and C, the reagents and laboratory procedure for INR measurement had changed. We suspected systematic overestimation of INR values in period A, especially for assays performed during the nightshift. Indeed, no statistically significant difference was observed for prothrombin rate, factor V, or fibrinogen values between the 3 periods.

# **Supplementary table 3: Clinical, biological course and prognosis of patients during the stay in intensive care unit according to the inclusion period**

|  | | | | **Periods** | | |  |
| --- | --- | --- | --- | --- | --- | --- | --- |
| **Variables** | | | **Total (n = 199)** | **A (n = 68)** | **B (n = 70)** | **C (n = 61)** | **P value** |
| **Worst biological values during ICU stay (until transplantation for those transplanted)** | | |  | | | | |
|  | Prothrombin time (%) | | 12 (5-18) | 12 (9-16) | 14 (5-20) | 10 (5-16) | 0.0524 |
|  | INR* | | 7.9 (4.9-12.7) | 10.3 (6.9-16.9) | 5.9 (3.8-10.6) | 7.9 (4.7-12) | < 0.0001 |
|  | Factor V (%) | | 12 (7-18) | 12 (7-19) | 12 (5-18) | 12 (8-18) | 0.8466 |
|  | Fibrinogen (g/L) | | 1.1 (0.7-1.6) | 1.1 (0.7-1.4) | 1.2 (0.6-1.5) | 1.3 (0.9-1.9) | 0.0733 |
|  | Phosphorus (mmol/L) | | 0.56 (0,33-1) | 0.47 (0.32-0.79) | 0.46 (0.32-1.04) | 0.71 (0.43-1.14) | 0.0124 |
|  | Ammonium (µmol/L) | | 142 (100-208) | 142 (103-194) | 140 (86-250) | 148 (112-211) | 0.6514 |
|  | Creatinine (µmol/L) | | 228 (99-429) | 242 (97-479) | 232 (149-418) | 216 (96-376) | 0.6889 |
|  | Lactate (mmol/L) | | 7.8 (4-13.3) | 6 (3.5-14.2) | 7.9 (3.7-13) | 8.2 (4.7-12.9) | 0.5304 |
|  | pH | | 7.31 (7.14-7.41) | 7.37 (7.21-7.42) | 7.31 (7.13-7.38) | 7.28 (7.11-7.38) | 0.0413 |
|  | Bicarbonate (mmol/L) | | 18 (12-23) | 19 (13-24) | 18 (12-22) | 17 (12-21) | 0.2547 |
|  | ASAT (UI/L) | | 6290 (2217-11655) | 5713 (2120-11736) | 7474 (2714-13044) | 5928 (2212-11261) | 0.4023 |
|  | ALAT (UI/L) | | 4090 (2232-6866) | 3680 (2155-5305) | 4527 (2437-8405) | 41433 (2245-6735) | 0.1768 |
|  | Total bilirubin (µmol/L) | | 173 (92-340) | 196 (103-368) | 160 (94-352) | 172 (71-261) | 0.2258 |
|  | Conjugated bilirubin (µmol/L) | | 106 (56-241) | 130 (61-241) | 83 (53-260) | 119 (53-212) | 0.467 |
|  | LDH (UI/L) | | 5120 (1381-10873) | 5460 (1704-10877) | 8638 (2142-15400) | 3074 (801-7664) | 0.0011 |
|  | MELD score* | | 47 (39-54) | 50 (44-58) | 46 (38-53) | 42 (37-51) | 0.0012 |
| Mechanical ventilation (no. (%)) | | | 128 (64.3) | 40 (58.8) | 45 (64.3) | 43 (70.5) | 0.3853 |
| Renal replacement therapy (no. (%)) | | | 102 (51.3) | 34 (50) | 41 (58.6) | 27 (44.3) | 0.2545 |
| Vasopressors (no. (%)) | | | 105 (52.8) | 30 (44.1) | 39 (55.7) | 36 (59) | 0.1978 |
| Bacterial infection (no. (%)) | | | 50 (25.1) | 17 (25) | 22 (31.4) | 11 (18) | 0.2112 |
| **Treatments** | | |  | | | | |
|  | N-acetyl-cysteine (no. (%)) | | 161 (80.9) | 43 (63.2) | 60 (85.7) | 58 (95.0) | < 0.0001 |
|  | Specific treatments regarding etiology (no. (%)) | | 105 (52.8) | 22 (32.4) | 39 (55.7) | 44 (72.1) | < 0.0001 |
| **Extracorporeal liver support** | | |  | | | | |
|  | MARS (no. (%)) | | 74 (37.2) | 34 (50.0) | 40 (57.1) | 0 (0.0) |  |
|  |  | MARS ≥ 17h (no. (%)) | 28 (18.2) | 17 (25.0) | 11 (15.7) | 0 (0.0) |  |
|  |  | MARS < 17h (no. (%)) | 46 (29.9) | 17 (25.0) | 29 (41.4) | 0 (0.0) |  |
|  | HVPE (no. (%)) | | 45 (22.6) | 0 (0.0) | 0 (0.0) | 45 (73.8) |  |
|  | No liver support (no. (%)) | | 80 (40.2) | 34 (50.0) | 30 (42.9) | 16 (26.2) |  |
| **Clinical course** | | | | | | | |
| O’Grady classification | | | | | | | 0.8032 |
|  | Hyperacute | | 167 (83.9) | 56 (82.4) | 60 (85.7) | 51 (83.6) |  |
|  | Acute | | 20 (10.1) | 6 (8.8) | 7 (10.0) | 7 (11.5) |  |
|  | Subacute | | 12 (6.0) | 6 (8.8) | 3 (4.3) | 3 (4.9) |  |
| Contraindication to LTx (no. (%)) | | | 63 (31.7) | 14 (20.6) | 26 (37.1) | 23 (37.7) | 0.535 |
| Registration on the waiting list (no. (%)) | | | 83 (41.7) | 44 (64.7) | 21 (30.0) | 18 (29.5) | < 0.0001 |
| Liver transplantation at day 21 | | | 44 (22.1) | 20 (29.4) | 13 (18.6) | 11 (18.0) | 0.2016 |
| **Transplant outcome and survival** | | |  | | | | |
|  | Listed, not transplanted, dead at day 21 | | 6 (3.0) | 3 (4.4) | 2 (2.9) | 1 (1.6) |  |
|  | Listed, not transplanted, alive at day 21 | | 22 (11.1) | 15 (22.1) | 4 (5.7) | 3 (4.9) |  |
|  | Listed, then unlisted due to contraindication, dead at day 21 | | 10 (5) | 5 (7.4) | 2 (2.9) | 3 (4.9) |  |
|  | Listed, then unlisted due to contraindication, alive at day 21 | | 1 (0.5) | 1 (1.5) | 0 (0.0) | 0 (0.0) |  |
|  | Listed, transplanted, dead at day 21 | | 9 (4.5) | 3 (4.4) | 4 (5.7) | 2 (3.3) |  |
|  | Listed, transplanted, alive at day 21 | | 35 (17.6) | 17 (25.0) | 9 (12.9) | 9 (14.8) |  |
|  | Not listed due to contraindication, dead at day 21 | | 44 (22.1) | 10 (14.7) | 19 (27.1) | 15 (24.6) |  |
|  | Not listed due to contraindication, alive at day 21 | | 22 (11.1) | 7 (10.3) | 7 (10.0) | 8 (13.1) |  |
|  | Not listed, no contraindication, alive at day 21 | | 50 (25.1) | 7 (10.3) | 23 (32.9) | 20 (32.8) |  |
| **Survival** | | |  | | | | |
|  | Transplant-free survival at day 21 | | 95 (47.7) | 30 (44.1) | 34 (48.6) | 31 (50.8) | 0.7375 |
|  | Overall survival at day 28 | | 132 (66.3) | 48 (70.6) | 43 (61.4) | 41 (67.2) | 0.5152 |
| ICU length of stay (days) | | | 6 (4-11) | 8 (4-13) | 6 (3-8) | 7 (4-11) | 0.0415 |

Data are expressed as median (25^th-^75^th^ percentiles) or numbers (%). Continuous variables are compared by the Mann-Whitney U test or the Kruskal-Wallis test and categorical variables are compared by the χ² test or the Fisher’s exact test. A P value < 0.05 is considered significant.

ICU: Intensive Care Unit, INR: International Normalized Ratio, ASAT: Aspartate aminotransferase, ALAT: Alanine aminotransferase, LDH: Lactate Dehydrogenase, MELD: Model for End stage Liver Disease, MARS: Molecular Adsorbent Recirculating System, HVPE: High Volume Plasma Exchange, LTx: Liver Transplantation.

* In periods B and C, the reagents and laboratory procedure for INR measurement had changed. We suspected systematic overestimation of INR values in period A, especially for assays performed during the nightshift. Indeed no statistically significant difference was observed for prothrombin rate, factor V, or fibrinogen values between the 3 periods. **Supplementary table 4: Baseline characteristics in intensive care unit and outcomes among the 138 patients during periods A and B according to the use of MARS dialysis**

|  | | | **MARS dialysis** | |  |
| --- | --- | --- | --- | --- | --- |
| **Variables** | | **Total (n = 138)** | **Yes (n = 74)** | **No (n = 64)** | **P value** |
| **Baseline characteristics** | | | | | |
| Sex H/F (%) | | 67/71 (48.6/51.4) | 31/43 (41.9/58.1) | 36/28 (56.3/43.8) | 0.0924 |
| Age (years) | | 46 (35-56) | 43 (33-55) | 49 (36-57) | 0.3859 |
| SOFA score | | 8 (5-13) | 8 (4-12) | 10 (6-14) | 0.0286 |
|  | Respiration SOFA | 0 (0-1) | 0 (0-1) | 1 (0-2) | 0.0239 |
|  | Coagulation SOFA | 1 (0-2) | 1 (0-2) | 2 (1-3) | 0.0159 |
|  | Liver SOFA | 2 (2-3) | 2 (2-3) | 2 (2-4) | 0.0913 |
|  | Cardiovascular SOFA | 0 (0-1) | 0 (0-1) | 0 (0-1) | 0.4136 |
|  | Central nervous system SOFA | 1 (0-3) | 1 (0-3) | 1 (0-3) | 0.9353 |
|  | Renal SOFA | 2 (0-4) | 3 (0-4) | 2 (0-4) | 0.3932 |
| SAPS II score | | 51 (31-68) | 47 (31-68) | 54 (33-67) | 0.4409 |
| Glasgow Coma Scale | | 14 (9-15) | 14 (9-15) | 14 (9-15) | 0.627 |
| MAP (mmHg) | | 86 (71-98) | 87 (74-98) | 84 (68-97) | 0.3356 |
| Heart rate (beats/min) | | 105 (90-120) | 103 (90-120) | 105 (90-120) | 0.721 |
| INR | | 5.2 (3.7-9.9) | 5.2 (3.7-9.7) | 5.2 (3.4-10) | 0.4918 |
| Creatinine (µmol/L) | | 162 (77-295) | 151 (75-295) | 192 (81-296) | 0.6929 |
| Lactate (mmol/L) | | 4.7 (2.6-9.7) | 5 (2.7-9.6) | 4.7 (2.5-9.8) | 0.9683 |
| ASAT (UI/L) | | 4792 (1525-10766) | 4746 (1922-10400) | 5812 (1128-10828) | 0.8678 |
| ALAT (UI/L) | | 3144 (1404-5127) | 2890 (1816-4910) | 3787 (841-6952) | 0.8376 |
| Total bilirubin (µmol/L) | | 89 (47-159) | 87 (49-139) | 95 (46-170) | 0.6447 |
| MELD score | | 38 (31-46) | 38 (31-47) | 39 (30-44) | 0.8176 |
| Medical history (no. (%)) | |  | | | |
|  | Diabetes mellitus | 5 (3.6) | 3 (4.1) | 2 (3.1) | 1 |
|  | Obesity | 11 (8.0) | 6 (8.1) | 5 (7.8) | 0.949 |
|  | Cardiovascular disease | 17 (12.3) | 10 (13.5) | 7 (10.9) | 0.6461 |
|  | Immunosuppression | 27 (19.6) | 14 (18.9) | 13 (20.3) | 0.837 |
| Mechanical ventilation during the first 24 hours (no. (%)) | | 70 (50.7) | 36 (48.6) | 34 (53.1) | 0.5999 |
| Renal replacement therapy during the first 24 hours (no. (%)) | | 58 (42.0) | 30 (40.5) | 28 (43.8) | 0.7033 |
| Vasopressors during the first 24 hours (no. (%)) | | 51 (37.0) | 27 (36.5) | 24 (37.5) | 0.9021 |
| HE grade (West-Haven criteria) | |  |  |  | 0.5023 |
|  | 0 | 25 (18.1) | 16 (21.6) | 9 (14.1) |  |
|  | 1 - 2 | 46 (33.3) | 23 (31.1) | 23 (35.9) |  |
|  | 3 - 4 (or intubation) | 67 (48.6) | 35 (47.3) | 32 (50.0) |  |
| Worsening of HE | | 61 (44.2) | 35 (47.3) | 26 (40.6) | 0.1617 |
| ALF etiologies | |  |  |  | 0.3483 |
|  | Acetaminophen | 51 (37.0) | 30 (40.5) | 21 (32.8) |  |
|  | Others | 87 (63.0) | 44 (59.5) | 43 (67.2) |  |
| **Clinical and biological course** | | | | | |
| Mechanical ventilation during the ICU stay (no. (%)) | | 85 (61.6) | 48 (64.9) | 37 (57.8) | 0.3956 |
| Renal replacement therapy during the ICU stay (no. (%)) | | 75 (54.3) | 43 (58.1) | 32 (50.0) | 0.3403 |
| Vasopressors during the ICU stay (no. (%)) | | 69 (50.0) | 37 (50.0) | 32 (50.0) | 1 |
| **Worst biological values during ICU stay (until transplantation for those transplanted)** | |  | | | |
|  | INR | 7.9 (5-13.4) | 8.8 (5.4-16) | 7 (4.5-11.6) | 0.1117 |
|  | Creatinine (µmol/L) | 234 (102-438) | 246 (102-429) | 222 (95-467) | 0.9421 |
|  | Total bilirubin (µmol/L) | 177 (96-360) | 187 (103-410) | 161 (83-325) | 0.2336 |
|  | ASAT (UI/L) | 6680 (2269-12294) | 6831 (3050-11300) | 6171 (1720-12776) | 0.5955 |
|  | ALAT (UI/L) | 4044 (2231-7033) | 4003 (2470-6716) | 4044 (1296-7233) | 0.7215 |
|  | Lactate (mmol/L) | 6.6 (3.5-13.6) | 7.9 (3.3-13.6) | 5.9 (3.8-13.5) | 0.9686 |
|  | pH | 6.32 (7.16-7.41) | 7.33 (7.16-7.4) | 7.32 (7.12-7.44) | 0.9095 |
| Total duration of MARS ≥ 17 hours (no. (%)) | | 28 (20.3) | 28 (37.8) | 0 (0.0) | < 0.0001 |
| Use of N-acetyl-cysteine (no. (%)) | | 103 (74.6) | 60 (81.1) | 43 (67.2) | 0.0614 |
| **Clinical course** | |  | | | |
|  | Contraindication to LTx (no. (%)) | 42 (30.4) | 24 (32.4) | 18 (28.1) | 0.5834 |
|  | Registration on the waiting list (no. (%)) | 65 (47.1) | 37 (50.0) | 28 (43.8) | 0.4332 |
| **Survival** | |  | | | |
|  | Transplant-free survival at day 21 | 64 (46.4) | 36 (48.6) | 28 (43.8) | 0.5650 |
|  | Liver transplantation at day 21 | 33 (23.9) | 19 (25.7) | 14 (21.9) | 0.6017 |
|  | Overall survival at day 28 | 91 (65.9) | 51 (68.9) | 40 (62.5) | 0.4275 |

Data are expressed as median (25^th-^75^th^ percentiles) or numbers (%). Continuous variables are compared by the Mann-Whitney U test or the Kruskal-Wallis test and categorical variables are compared by the χ² test or the Fisher’s exact test. A P value < 0.05 is considered significant.

ICU: Intensive Care Unit, MARS: Molecular Adsorbent Recirculating System, SOFA: Sequential Organ Failure Assessment, SAPS II: Simplified Acute Physiology Score II, MAP: Mean Arterial Pressure, INR: International Normalized Ratio, ASAT: Aspartate aminotransferase, ALAT: Alanine aminotransferase, MELD: Model for End stage Liver Disease, HE: Hepatic Encephalopathy, ALF: Acute Liver Failure, LTx: Liver Transplantation.

# **Supplementary table 5: Baseline characteristics in intensive care unit and outcomes among 74 patients treated with MARS during periods A and B according to the duration of MARS therapy**

|  | | | **MARS therapy** | |  |
| --- | --- | --- | --- | --- | --- |
| **Variables** | | **Total (n = 74)** | **< 17 hours (n = 46)** | **≥ 17 hours (n = 28)** | **P value** |
| **Baseline characteristics** | | | | | |
| Sex H/F (%) | | 31/43 (41.9/58.1) | 17/29 (37/63) | 14/14 (50/50) | 0.2701 |
| Age (years) | | 43 (33-55) | 47 (33-47) | 43 (34-51) | 0.2719 |
| SOFA score | | 8 (4-12) | 8 (4-13) | 8 (5-10) | 0.5723 |
| SAPS II score | | 47 (31-68) | 50 (32-72) | 39 (31-58) | 0.2264 |
| Glasgow Coma Scale | | 14 (9-15) | 14 (7-15) | 14 (12-15) | 0.4534 |
| MAP (mmHg) | | 87 (74-98) | 83 (71-98) | 90 (79-102) | 0.1634 |
| Heart rate (beats/min) | | 103 (90-120) | 103 (90-115) | 103 (91-125) | 0.5135 |
| INR | | 5.2 (3.7-9.7) | 5 (3.7-10) | 5.5 (3.8-8) | 0.7977 |
| Creatinine (µmol/L) | | 151 (75-295) | 200 (82-357) | 109 (75-231) | 0.1296 |
| Lactate (mmol/L) | | 5 (2.7-9.6) | 5.5 (2.9-9.6) | 4.4 (2.2-8.8) | 0.3611 |
| ASAT (UI/L) | | 4746 (1922-10400) | 5278 (1525-10400) | 4281 (2271-9980) | 0.9600 |
| ALAT (UI/L) | | 2890 (1816-4910) | 3260 (2289-5127) | 2543 (1116-4312) | 0.1368 |
| Total bilirubin (µmol/L) | | 87 (49-139) | 80 (44-129) | 98 (64-158) | 0.2308 |
| MELD score | | 38 (31-47) | 39 (32-50) | 36 (31-43) | 0.1979 |
| Medical history (no. (%)) | |  | | | |
|  | Diabetes mellitus | 3 (4.1) | 3 (6.5) | 0 (0.0) | 0.2847 |
|  | Obesity | 6 (8.1) | 5 (10.9) | 1 (3.6) | 0.3986 |
|  | Cardiovascular disease | 10 (13.5) | 8 (17.4) | 2 (7.1) | 0.3015 |
|  | Immunosuppression | 14 (18.9) | 6 (13.0) | 8 (28.6) | 0.0971 |
| Mechanical ventilation during the first 24 hours (no. (%)) | | 36 (48.6) | 23 (50.0) | 13 (46.4) | 0.7656 |
| Renal replacement therapy during the first 24 hours (no. (%)) | | 30 (40.5) | 21 (45.7) | 9 (32.1) | 0.2510 |
| Vasopressors during the first 24 hours (no. (%)) | | 27 (36.5) | 16 (34.8) | 11 (39.3) | 0.6963 |
| HE grade (West-Haven criteria) | |  |  |  | 0.5599 |
|  | 0 | 16 (21.6) | 9 (19.6) | 7 (25.0) |  |
|  | 1 - 2 | 23 (31.1) | 13 (28.3) | 10 (35.7) |  |
|  | 3 - 4 (or intubation) | 35 (47.3) | 24 (52.2) | 11 (39.3) |  |
| Worsening of HE | | 35 (47.3) | 23 (50.0) | 12 (42.9) | 0.2404 |
| ALF etiologies | |  |  |  | 0.8638 |
|  | Acetaminophen | 30 (40.5) | 19 (41.3) | 11 (39.3) |  |
|  | Others | 44 (59.5) | 27 (58.7) | 17 (60.7) |  |
| Number of MARS therapy sessions | | 2 (1-3) | 1 (1-2) | 3 (3-4) | < 0.0001 |
| Total duration of MARS therapy (hours) | | 15.5 (8-24) | 8 (7.5-13.5) | 24 (22.25-32) | < 0.0001 |
| **Clinical and biological course** | | | | | |
| Mechanical ventilation during the ICU stay (no. (%)) | | 48 (64.9) | 29 (63.0) | 19 (67.9) | 0.6740 |
| Renal replacement therapy during the ICU stay (no. (%)) | | 43 (58.1) | 26 (65.5) | 17 (60.7) | 0.7230 |
| Vasopressors during the ICU stay (no. (%)) | | 37 (50.0) | 24 (52.2) | 13 (46.4) | 0.6317 |
| **Worst biological values during ICU stay (until transplantation for those transplanted)** | |  | | | |
|  | INR | 8.8 (5,4-16) | 9.7 (5.9-16) | 7.7 (5.3-13.7) | 0.7677 |
|  | Creatinine (µmol/L) | 246 (102-429) | 259 (140-444) | 207 (101-428) | 0.4255 |
|  | Total bilirubin (µmol/L) | 187 (103-410) | 131 (94-366) | 254 (160-442) | 0.0063 |
|  | ASAT (UI/L) | 6831 (3050-11300) | 6681 (2891-10958) | 7320 (3836-13524) | 0.6437 |
|  | ALAT (UI/L) | 4003 (2470-6716) | 4003 (2571-7900) | 3838 (1963-5268) | 0.3078 |
|  | Lactate (mmol/L) | 7.9 (3.3-13.6) | 7.9 (3.3-13.6) | 7.3 (3.2-13.8) | 0.8366 |
|  | pH | 7.33 (7.16-7.4) | 7.31 (7.15-7.4) | 7.34 (7.21-7.41) | 0.2894 |
| Use of N-acetyl-cysteine (no. (%)) | | 60 (81.1) | 37 (80.4) | 23 (82.1) | 0.8556 |
| **Clinical course** | |  | | | |
|  | Contraindication to LTx (no. (%)) | 22 (29.7) | 9 (19.6) | 13 (46.4) | 0.0142 |
|  | Registration on the waiting list (no. (%)) | 37 (50.0) | 28 (60.9) | 9 (32.1) | 0.0165 |
| **Survival** | |  | | | |
|  | Transplant-free survival at day 21 | 36 (48.6) | 14 (30.4) | 22 (78.6) | < 0.0001 |
|  | Liver transplantation at day 21 | 19 (25.7) | 18 (39.1) | 1 (3.6) | 0.0007 |
|  | Overall survival at day 28 | 51 (68.9) | 28 (60.9) | 23 (82.1) | 0.0552 |

Data are expressed as median (25^th-^75^th^ percentiles) or numbers (%). Continuous variables are compared by the Mann-Whitney U test or the Kruskal-Wallis test and categorical variables are compared by the χ² test or the Fisher’s exact test. A P value < 0.05 is considered significant.

ICU: Intensive Care Unit, MARS: Molecular Adsorbent Recirculating System, SOFA: Sequential Organ Failure Assessment, SAPS II: Simplified Acute Physiology Score II, MAP: Mean Arterial Pressure, INR: International Normalized Ratio, ASAT: Aspartate aminotransferase, ALAT: Alanine aminotransferase, MELD: Model for End stage Liver Disease, HE: Hepatic Encephalopathy, ALF: Acute Liver Failure, LTx: Liver Transplantation.

# **Supplementary table 6: Univariate and multivariate analysis of Day-21 transplant-free survival among 74 patients treated with MARS during periods A and B**

|  | **Univariate analysis** | | **Multivariate analysis** | |
| --- | --- | --- | --- | --- |
| **Variables** | **HR [CI 95%]** | **P value** | **HR [IC 95%]** | **P value** |
| Obesity | 3.50 [1.44-8.52] | 0.0058 |  |  |
| Acetaminophen or toxic etiology | 0.44 [0.23-0.84] | 0.0131 |  |  |
| SOFA score > 8 at admission | 1.26 [0.66-2.38] | 0.4856 |  |  |
| SAPS II > 50 at admission | 1.79 [0.95-3.40] | 0.0726 |  |  |
| MELD > 38 at admission | 2.45 [1.25-4.81] | 0.0094 | 2.38 [1.15-4.93] | 0.0192 |
| Grade 3 - 4 (or intubation) HE at admission | 1.09 [0.50-2.40] | 0.3991 |  |  |
| Need for vasopressors during the first 24 hours | 1.75 [0.92-3.33] | 0.0866 | 2.13 [1.06-4.26] | 0.0336 |
| Need for mechanical ventilation during the first 24 hours | 1.54 [0.81-2.92] | 0.1874 |  |  |
| Need for renal replacement therapy during the first 24 hours | 1.56 [0.83-2.96] | 0.1709 |  |  |
| Improvement of HE after admission | 0.23 [0.08-0.64] | 0.0051 | 0.25 [0.09-0.73] | 0.0110 |
| INR > 6,5 at admission | 1.96 [1.03-3.72] | 0.0405 |  |  |
| Lactate > 5 mmol/L at admission | 2.37 [1.20-4.69] | 0.0127 |  |  |
| Use of MARS therapy ≥ 17 hours | 0.20 [0.08-0.49] | 0.0004 | 0.18 [0.07-0.46] | 0.0003 |
| Immunosuppressive therapy during the past year | 1.64 [0.68-3.92] | 0.2671 |  |  |
| Use of N-acetyl-cysteine | 0.40 [0.20-0.82] | < 0.0001 | 0.33 [0.16-0.69] | 0.0032 |

For multivariate analysis, a Cox model with a stepwise backward selection has been used. The results are reported as Hazard Ratio (HR) and the 95% confidence interval (95% CI).

MARS: Molecular Adsorbent Recirculating System, SOFA: Sequential Organ Failure Assessment, SAPS II: Simplified Acute Physiology Score II, MELD: Model for End stage Liver Disease, HE: Hepatic Encephalopathy, INR: International Normalized Ratio.

# **Supplementary table 7: Baseline characteristics in intensive care unit and outcomes in 45 patients treated with HVPE and 126 controls**

| **Variables** | | **Total (n = 171)** | **HVPE (n = 45)** | **Control group (n = 126)** | **P value** |
| --- | --- | --- | --- | --- | --- |
| **Baseline characteristics** | | | | | |
| Sex H/F (%) | | 81/90 (47.4/52.6) | 19/26 (42.2/57.8) | 62/64 (49.2/50.8) | 0.4206 |
| Age (years) | | 47 (36-58) | 49 (36-59) | 47 (36-57) | 0.6097 |
| SOFA score | | 8 (5-12) | 7 (4-9) | 9 (5-13) | 0.0143 |
| SAPS II score | | 49 (31-65) | 43 (31-55) | 53 (31-68) | 0.0665 |
| SAPS II score > 47 at admission (no. (%)) | | 88 (51.5) | 18 (40) | 70 (55.6) | 0.0731 |
| Glasgow Coma Scale | | 14 (8-15) | 14 (11-15) | 14 (6-15) | 0.3250 |
| MAP (mmHg) | | 82 (69-97) | 86 (72-97) | 82 (69-96) | 0.3910 |
| Heart rate (beats/min) | | 105 (90-115) | 105 (97-110) | 105 (90-120) | 0.6139 |
| INR | | 5.2 (3.4-9.8) | 6.6 (4.6-9.8) | 4.9 (3.2-9.7) | 0.1161 |
| Lactate (mmol/L) | | 5 (2.7-9.6) | 5.1 (2.8-10) | 5 (2.7-9.3) | 0.9396 |
| ASAT (UI/L) | | 4385 (1389-10400) | 2847 (1187-9054) | 5398 (1436-10667) | 0.2130 |
| ALAT (UI/L) | | 3644 (1445-5330) | 4134 (1430-5057) | 3537 (1547-5330) | 0.7231 |
| Total bilirubin (µmol/L) | | 85 (44-158) | 87 (44-119) | 85 (44-159) | 0.6712 |
| MELD score | | 38 (29-44) | 38 (32-42) | 38 (29-45) | 0.6011 |
| Medical history (no. (%)) | |  | | | |
|  | Diabetes mellitus | 9 (5.3) | 2 (4.4) | 7 (5.6) | 1 |
|  | Obesity | 13 (7.6) | 2 (4.4) | 11 (8.7) | 0.5176 |
|  | Cardiovascular disease | 20 (11.7) | 3 (6.7) | 17 (13.5) | 0.2213 |
|  | Immunosuppression | 23 (13.5) | 4 (8.9) | 19 (15.1) | 0.2213 |
|  | Immunosuppressive therapy during the past year | 12 (7.0) | 0 (0.0) | 12 (9.5) | 0.0376 |
| Mechanical ventilation during the first 24 hours (no. (%)) | | 88 (51.5) | 16 (35.6) | 47 (37.3) | 0.8349 |
| Renal replacement therapy during the first 24 hours (no. (%)) | | 62 (36.3) | 8 (17.8) | 54 (42.9) | 0.0027 |
| Vasopressors during the first 24 hours (no. (%)) | | 63 (36.8) | 16 (35.6) | 47 (37.3) | 0.8349 |
| HE grade (West-Haven criteria) | |  |  |  | 0.0290 |
|  | 0 | 28 (22.2) | 16 (35.6) | 22 (17.5) |  |
|  | 1 - 2 | 54 (31.6) | 14 (31.1) | 40 (31.7) |  |
|  | 3 - 4 (or intubation) | 79 (46.2) | 15 (33.3) | 64 (50.8) |  |
| Worsening of HE | | 74 (43.3) | 21 (46.7) | 53 (42.1) | 0.3625 |
| ALF etiologies | |  |  |  | 0.1283 |
|  | Acetaminophen | 71 (41.5) | 23 (51.1) | 48 (38.1) |  |
|  | Others | 100 (58.5) | 22 (48.9) | 78 (61.9) |  |
| **Clinical and biological course** | | | | | |
| Mechanical ventilation during the ICU stay (no. (%)) | | 109 (63.7) | 32 (71.1) | 77 (61.1) | 0.2310 |
| Renal replacement therapy during the ICU stay (no. (%)) | | 85 (49.7) | 19 (42.2) | 66 (52.4) | 0.2420 |
| Vasopressors during the ICU stay (no. (%)) | | 92 (53.8) | 26 (57.8) | 66 (52.4) | 0.5331 |
| Maximum calculable SOFA score* | | 15 (8-20) | 16 (12-21) | 13 (7-20) | 0.0335 |
| **Worst biological values during ICU stay (until transplantation for those transplanted)** | |  | | | |
|  | INR | 7.9 (4.7-12.7) | 8.4 (6.2-12) | 7.6 (4.4-13.3) | 0.7284 |
|  | Creatinine (µmol/L) | 229 (98-436) | 230 (90-351) | 227 (121-449) | 0.2579 |
|  | Total bilirubin (µmol/L) | 159 (81-302) | 175 (81-275) | 148 (87-324) | 0.9958 |
|  | ASAT (UI/L) | 6193 (2132-11389) | 5928 (1670-11226) | 6445 (2146-11608) | 0.5827 |
|  | ALAT (UI/L) | 4090 (2233-7033) | 4338 (2287-6852) | 3960 (2231-7200) | 0.7113 |
|  | Lactate (mmol/L) | 7.8 (4.1-13.2) | 7.8 (4.7-13) | 7.8 (3.8-13.5) | 0.3371 |
|  | pH | 7.3 (7.13-7.4) | 7.28 (7.12-7.39) | 7.31 (7.15-7.41) | 0.4265 |
| MARS therapy (no. (%)) | | 46 (26.9) | 0 (0.0) | 46 (36.5) | < 0.0001 |
| Use of N-acetyl-cysteine (no. (%)) | | 138 (80.7) | 44 (97.8) | 94 (74.6) | 0.0007 |
| **Clinical course** | |  | | | |
|  | Contraindication to LTx (no. (%)) | 50 (29.2) | 14 (31.1) | 36 (28.6) | 0.7478 |
|  | Registration on the waiting list (no. (%)) | 74 (43.3) | 16 (35.6) | 58 (46.0) | 0.2234 |
| **Survival** | |  | | | |
|  | Transplant-free survival at day 21 | 73 (42.7) | 25 (55.6) | 48 (38.1) | 0.0421 |
|  | Liver transplantation at day 21 | 43 (25.1) | 10 (22.2) | 33 (26.2) | 0.5984 |
|  | Overall survival at day 28 | 109 (63.7) | 34 (75.6) | 75 (59.5) | 0.548 |

Data are expressed as median (25^th-^75^th^ percentiles) or numbers (%). Continuous variables are compared by the Mann-Whitney U test or the Kruskal-Wallis test and categorical variables are compared by the χ² test or the Fisher’s exact test. A P value < 0.05 is considered significant.

ICU: Intensive Care Unit, HVPE: High Volume Plasma Exchange, SOFA: Sequential Organ Failure Assessment, SAPS II: Simplified Acute Physiology Score II, MAP: Mean Arterial Pressure, INR: International Normalized Ratio, ASAT: Aspartate aminotransferase, ALAT: Alanine aminotransferase, MELD: Model for End stage Liver Disease, HE: Hepatic Encephalopathy, ALF: Acute Liver Failure, LTx: Liver Transplantation. *Calculated by adding the highest value of each sub-score (respiration, coagulation, liver, cardiovascular, central nervous system, renal) measured every day from ICU admission to ICU discharge or transplantation.

#
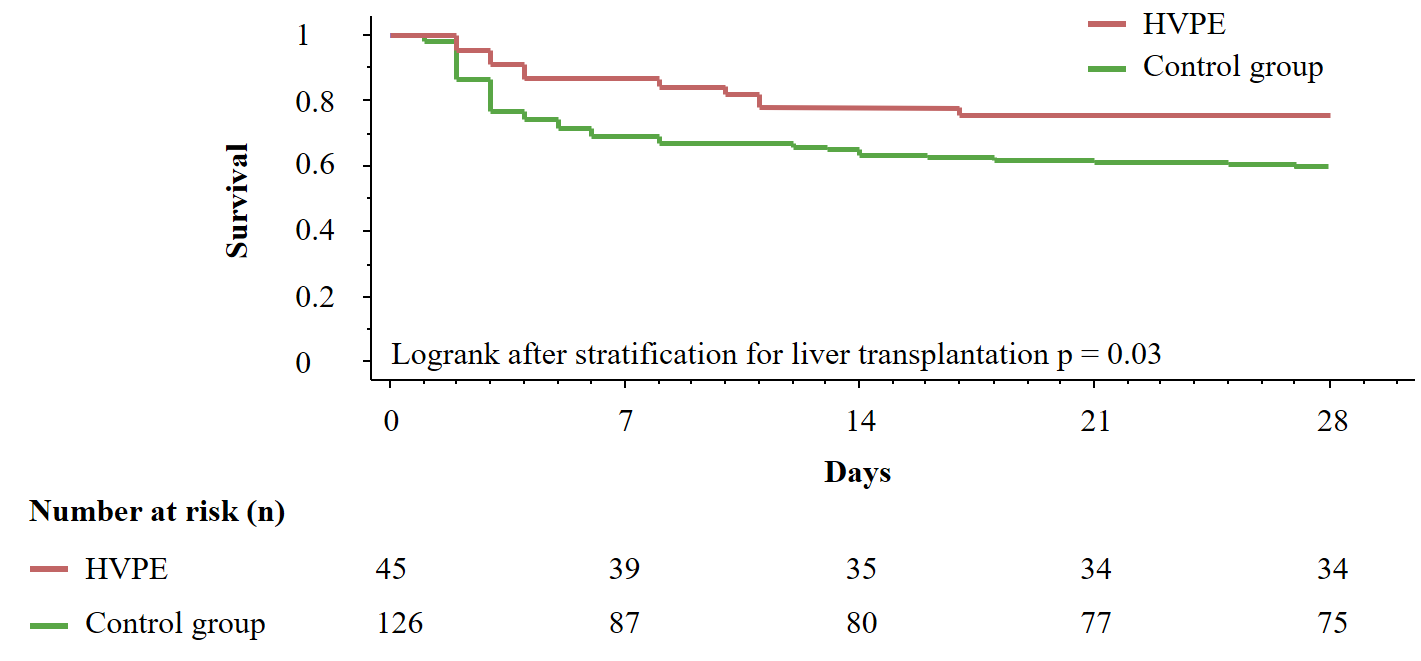
**Supplementary figure 1: Day-28 overall survival in 45 patients treated with HVPE and in 126 controls**

HVPE: High Volume Plasma Exchange.

#
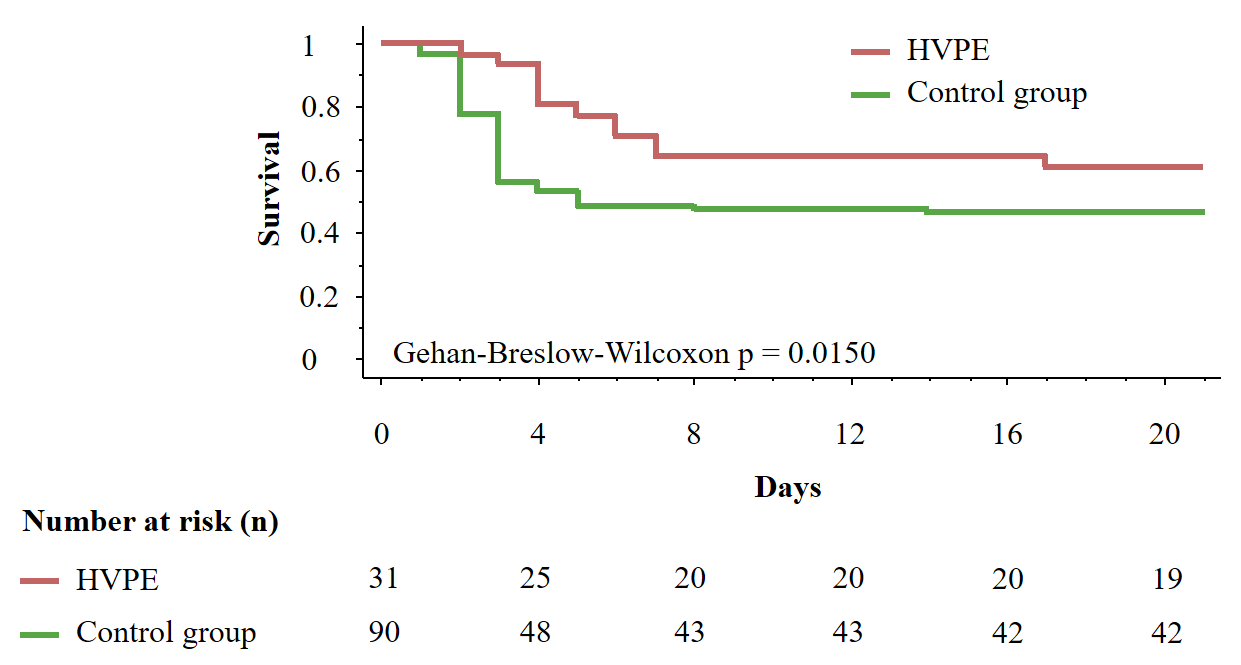
**Supplementary figure 2: Day-21 transplant-free survival in 31 patients treated with HVPE and 90 controls without liver transplantation contraindications**

HVPE: High Volume Plasma Exchange.

# **
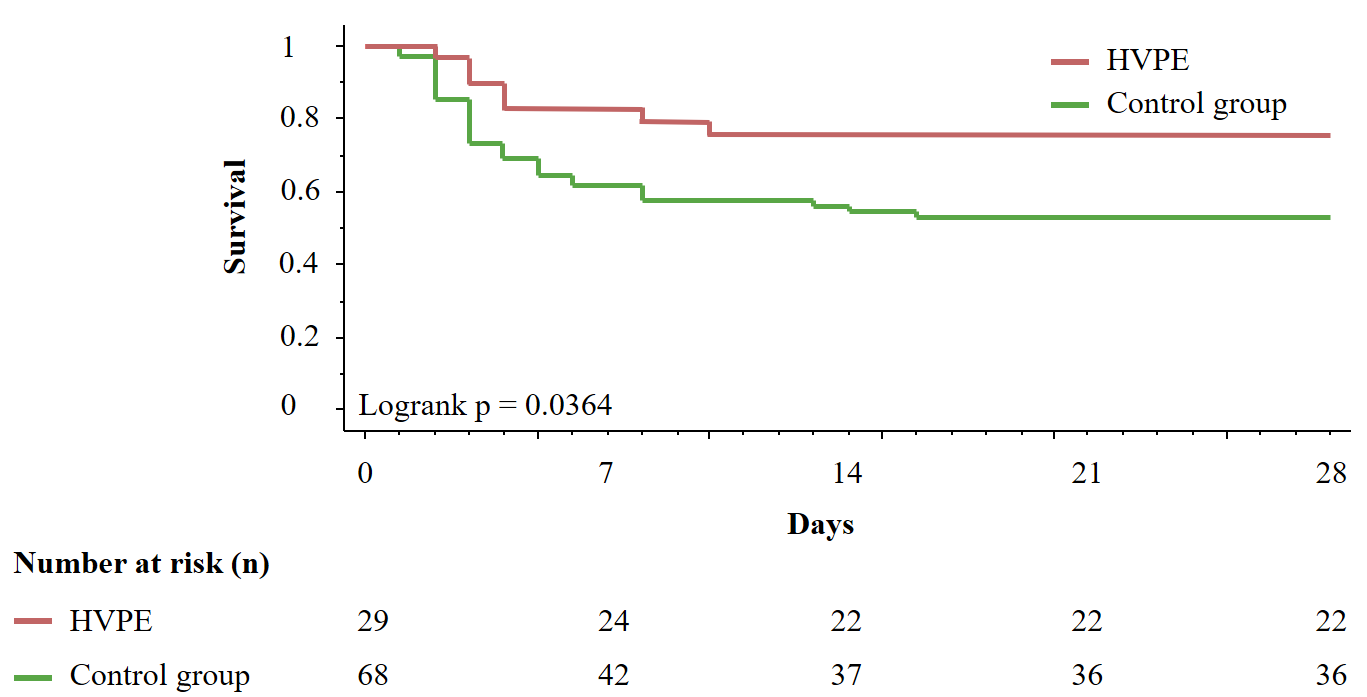
Supplementary figure 3: Day-28 survival among 29 patients treated with HVPE and 68 controls not listed for transplant**

HVPE: High Volume Plasma Exchange.

#
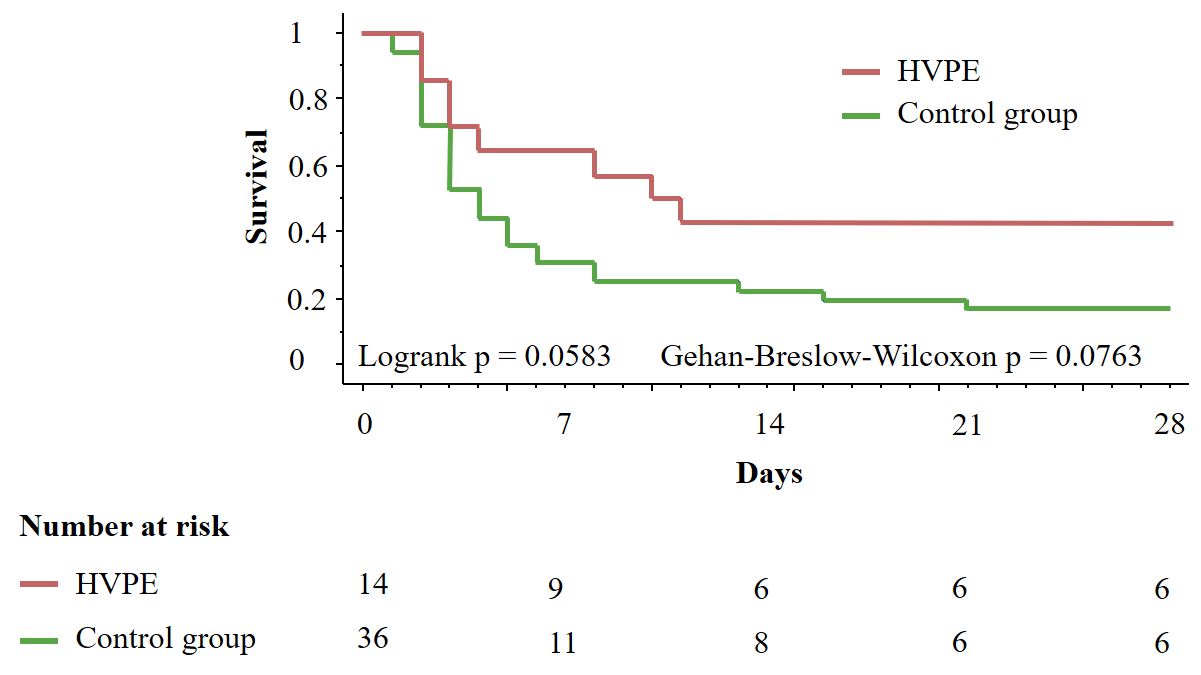
**Supplementary figure 4: Day-28 survival according to HVPE treatment or control among 50 patients not listed due to contraindications to liver transplantation**

HVPE: High Volume Plasma Exchange.
